# Human cleaving embryos enable efficient mitochondrial base-editing with DdCBE

**DOI:** 10.1038/s41421-021-00372-0

**Published:** 2022-02-01

**Authors:** Yinghui Wei, Chunlong Xu, Hu Feng, Kui Xu, Zhifang Li, Jing Hu, Ling Zhou, Yu Wei, Zhenrui Zuo, Erwei Zuo, Wen Li, Hui Yang, Meiling Zhang

**Affiliations:** 1 International Peace Maternity and Child Health Hospital, School of Medicine, Shanghai Jiao Tong University, Shanghai, China; 2 Institute of Neuroscience, State Key Laboratory of Neuroscience, Key Laboratory of Primate Neurobiology, CAS Center for Excellence in Brain Science and Intelligence Technology, Shanghai Institutes for Biological Sciences, Chinese Academy of Sciences, Shanghai, China; 3 Shanghai Center for Brain Science and Brain-Inspired Intelligence Technology, Shanghai, China; 4 Shenzhen Branch, Guangdong Laboratory for Lingnan Modern Agriculture, Genome Analysis Laboratory of the Ministry of Agriculture, Agricultural Genomics Institute at Shenzhen, Chinese Academy of Agricultural Sciences, Shenzhen, China

**Keywords:** Biological techniques, Cell biology

Dear Editor,

Mitochondrial diseases could be caused by heritable mutations in both mtDNA and nuclear DNA^1–3^. Because mitochondria membrane^4^ insulated mtDNAs’ access from large ribonucleoprotein complex of Cas9 and sgRNA, mtDNAs are intractable for genetic modifications with RNA-guided CRISPR/Cas9 system commonly used for effectively editing nuclear DNA of different species. Although ZFN and TALEN have been previously engineered to successfully cut and eliminate mtDNA in a programmable way^5–9^, correction of disease-causing point mutations in mtDNA remained challenging. Recently, DddA-derived cytosine base editors (DdCBEs) have been developed to specifically induce C-to-T conversion in mtDNA by fusion of sequence-programmable TALE and split deaminase derived from interbacterial toxins^10^. DdCBE has been demonstrated to generate mutant zebrafish and mice carrying base-modified mtDNA by injecting DdCBE mRNA into zygotes^11,12^. However, the efficacy of DdCBE for specific installation of C-to-T conversion in mtDNA of human embryos remained to be investigated.

In this study, we sought to test DdCBE’s ability for base editing of mtDNA in human embryos. We first designed a pair of TALE recognizing 34 bp sequences of *ND4* gene on mitochondrial genome. Split deaminase pair DddA_half_ G1397-C and G1397-N, along with SOD2/COX8A mitochondrial targeting signal and uracil glycosylase inhibitor, were further fused with the TALE pair respectively to generate *ND4*-DdCBE that targets *ND4* locus. After in vitro transcription, we injected the DdCBE mRNA into clinically discarded human embryos with three pronuclei (3PN) to check protein expression and cellular localization. Immunostaining results using anti-HA and anti-FLAG antibodies revealed high expression of the DddA-TALE fusion deaminase pairs and their proper co-localization with mitotracker signal in the blastomere cell of human embryos (Supplementary Fig. S1a), similar to the recent finding in HEK293T cells^10^. Our previous study with cytosine and adenine base editors in human embryos showed that cleaving embryos enable robust base conversion in nuclear DNA^13^. To determine the optimal embryonic stage for mtDNA editing, we injected *ND4*-DdCBE mRNA into human zygote, 2-cell, 4-cell and 8-cell embryos, respectively, and performed genotyping analysis 48 h post injection (Fig. 1a). We found that *ND4*-DdCBE exhibited detectable activity for C4 position, and injection of human embryos at the cleavage stage significantly improved base editing efficiency of *ND4*-DdCBE, compared with the injection at the zygote stage (Fig. 1b; Supplementary Fig. S1c). To confirm the finding with *ND4*-DdCBE, we further constructed DdCBE targeting *ND6*, *ND1*, *ND5.1*, and *ATP8* genes on mitochondrial genome. By injection of *ND6*-, *ND1*-, *ND5.1*-, or *ATP8*-DdCBE mRNA into human 3PN-deriverd zygote and cleaving embryos, we found DdCBE also induced efficient C-to-T base conversions and injection of DdCBE at 8-cell stage showed dramatic increase of the base conversion rate (Supplementary Figs. S1c and S2a–d). In particular, human embryos at 8-cell stage could support up to 60% cytosine conversion compared with less than 10% cytosine conversion at other stages (Supplementary Figs. S1c and S2a–d). Moreover, we showed that the advantage of using 8-cell embryos was not due to the total amount of injected editing agents, since when we increased the concentration of editing agents for 2-cell embryos by 4-fold and 4-cell embryos by 2-fold, no significant improvement of editing efficiency was observed (Supplementary Fig. S1b).

**Fig. 1:**
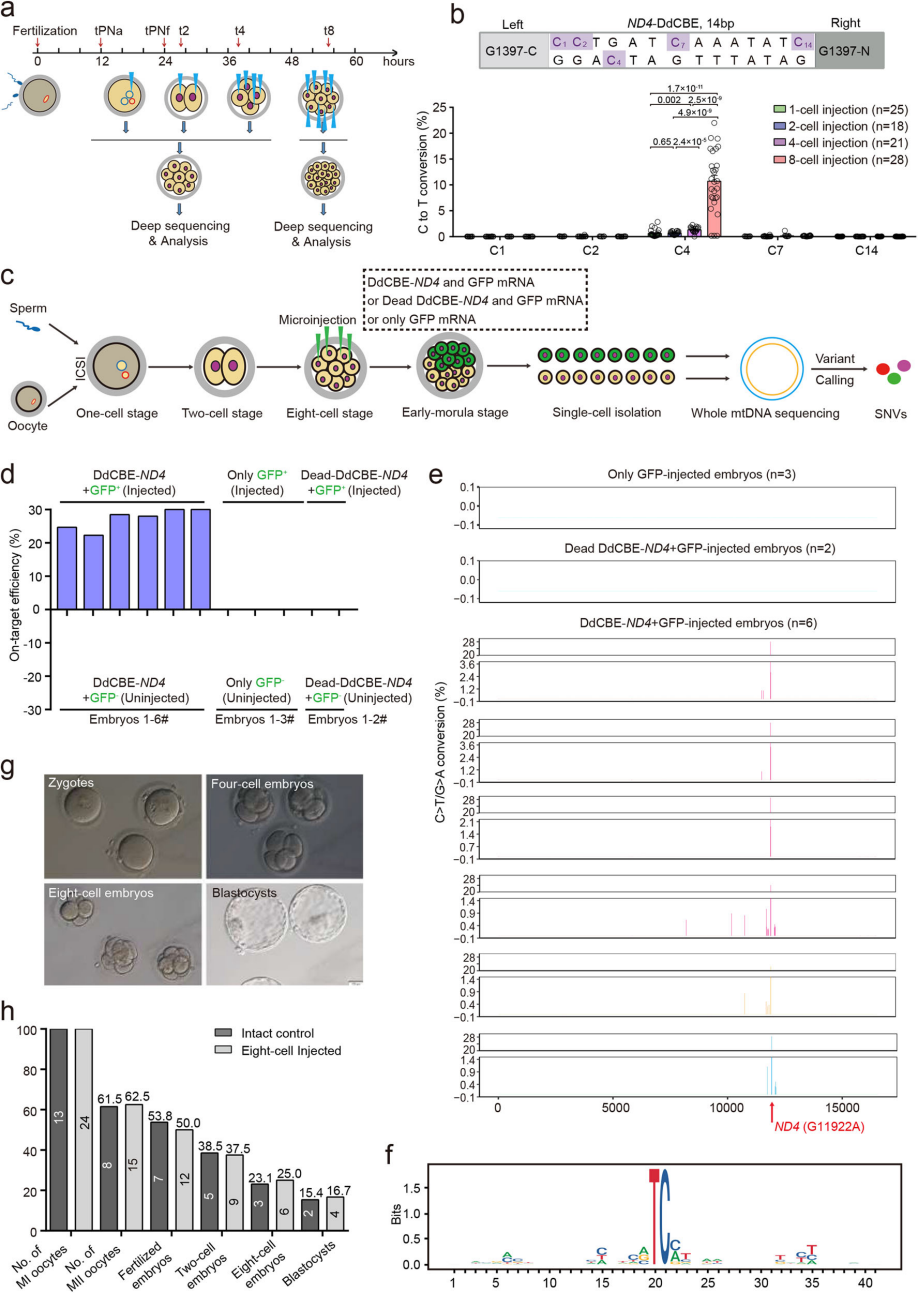
Human 8-cell embryos mediate robust mitochondrial base editing with DdCBE. **a** Experimental workflow of DdCBE injection in human embryos and genotyping analysis. **b** Percentage of total sequencing reads with C·G base pair converted to T·A in *ND4*-DdCBE-injected 1-cell, 2-cell, 4-cell, and 8-cell embryos of 3PN. **c** Experimental workflow of DdCBE injection for off-target profiling in human 2PN embryos. **d** On-target efficiency of *ND4*-DdCBE in human 2PN embryos. Embryos injected with *ND4*-Dead-DdCBE + *GFP* or only GFP mRNA showed no base editing on the *ND4* site. **e** Mitochondrial genome-wide off-target loci identified from human 2PN embryos injected with *ND4*-DdCBE. No SNV was identified in control embryos injected with *ND4*-Dead-DdCBE + *GFP* or *GFP* mRNA only whereas several SNVs were identified in embryos injected with both GFP and *ND4*-DdCBE mRNA, possibly due to off-target editing. **f** Sequence logos generated from sequences with off-target C·G to T·A conversions by *ND4*-DdCBE on mitochondrial DNA genome. Bits reflect sequence conservation at a given position. **g** Normal morphology of human 2PN embryos injected with *ND4*-DdCBE at different developmental stages. **h** Comparable developmental rate between uninjected human 2PN embryos and the ones injected with *ND4*-DdCBE. Data are presented as means ± SEM. *P* values were evaluated with the unpaired student’s *t*-test (two-tailed).

To further verify our finding from 3PN embryo experiments in normal 2PN embryos (derived from immature MI oocyte injected with sperm) and interrogate off-target effects of DdCBE, we performed mitochondrial genome-wide sequencing analysis after injecting *ND4*-DdCBE/Dead-DdCBE and *GFP* mRNA or only *GFP* mRNA into four blastomeres of 2PN 8-cell embryos (Fig. 1c). The other four blastomeres were left un-injected to eliminate the differences in the genetic background between the gene-edited and control embryos. We found only GFP-positive blastomeres (GFP^+^) injected with both *ND4-*DdCBE and *GFP* mRNA showed around 25% C•G-to-T•A conversions while dead DdCBE-injected, uninjected (GFP^−^), or GFP only ones remained intact on target loci (Fig. 1d). We then performed mtDNA sequencing of both GFP^+^ and GFP^−^ blastomeres, and used GFP^−^ blastomeres as a negative control to call single nucleotide variations (SNVs) potentially resulted from *ND4-*DdCBE off-target effects on mitochondrial genome. Most of SNVs identified in 2PN GFP^+^ embryos were centered around on-target loci (Fig. 1e), probably due to dsDNA affinity of deaminase derived from interbacterial toxins^10^ and unstable binding of target sequence by the TALE pair. Besides SNVs caused by bystander effects of DdCBE, there were some distal SNVs with less than 1% allelic frequency far from TALE recognition sequences (Fig. 1e), potentially caused by off-target activity of the DdCBE. To further characterize off-target profile of DdCBE, we performed the same experiments at mitochondrial *ND6* locus. Similar to the *ND4*-DdCBE results, *ND6-*DdCBE showed around 20% on-target C•G-to-T•A conversions (Supplementary Fig. S4a). However, *ND6-*DdCBE yielded more off-target SNVs than the *ND4*-DdCBE (Supplementary Fig. S4b), probably due to the difference of TALE recognition sequences. Furthermore, we analyzed all SNVs identified in *ND4*- and *ND6*-targeting samples and found significant enrichment of C-to-T/G-to-A conversion (Supplementary Figs. S3b and S4c). Besides, we retrieved the 20 bp regions flanking each off-target SNV, and found a strong 5’-TC-3’ preference for *ND4*- and *ND6*-DdCBE off-target edits (Fig. 1f; Supplementary Figs. S3a and S4d), consistent with the identity of cytosine deaminase for DdCBE^10^. In addition, we did not observe significant off-target editing in nuclear pseudogenes, even though they differ by only 0–1 bp from the target sites on mtDNA (Supplementary Fig. S5). Finally, we checked the morphology and developmental rate of human 2PN embryos after the injection of DdCBE mRNA. We detected no morphological abnormality of 8-cell injected embryos compared with normal ones (Fig. 1g). Development rate also showed non-significant difference between 8-cell injected embryos and non-injected ones, implying high applicability of DdCBE in human embryos for specific mtDNA modifications (Fig. 1h).

Taken together, our results demonstrate that DdCBE is an effective base editor for inducing point mutations in mtDNA of human embryos, and the efficiency is much higher in 8-cell embryos. Our 8-cell injection method could help generate mitochondrial disease models as well as derived embryonic stem cells for functional investigation of disease-associated mutations in mtDNA. The current study is performed on clinically discard 3PN and 2PN embryos from healthy people. Therefore, it warrants further study to test the capability of DdCBE to correct disease-causing mutations in mtDNA of the embryos from donor patients, which may provide the alternative way for prevention of heritable mtDNA mutations leading to the untreatable mitochondrial diseases after birth. Given the bystander and off-target editing profile, DdCBE remains to be further optimized for both basic and therapeutic research in the future.

## Supplementary information

Supplementary information
