# Supplementary material for: Human cleaving embryos enable efficient mitochondrial base-editing with DdCBE
Source: Cell Discov. 2022 Feb 1;8:7. doi: 10.1038/s41421-021-00372-0 (PMC8803867; doi:10.1038/s41421-021-00372-0)
Supplement: Supplementary file 1 — Supplementary information [file 41421_2021_372_MOESM1_ESM.pdf]

Supplementary information

Human cleaving embryos enable efficient mitochondrial base-editing with DdCBE

Contents

Ethics statement

Materials and Methods

Supplementary figures and legends

Supplementary Table S1

Supplementary sequence

Ethics statement

The regulatory framework for the use of human gametes and embryos in this research was based on the Management of Human Assisted Reproductive Technology (2001), Regulations of Human Assisted Reproductive Technology (2003), Human Biomedical Research Ethics Guidelines (set by National Health and Family Planning Commission of the People's Republic of China on Nov. 1st, 2020), the Human Embryonic Stem Cell Research Ethics Guidelines (2003), the 2021 Guidelines for Stem Cell Research and Clinical Translation (issued by the International Society for Stem Cell Research, ISSCR), the latest Heritable Human Genome Editing Report (issued by the International Commission on the Clinical Use of Human Germline Genome Editing on Sep. 3rd, 2020), the Helsinki Declaration, and other laws and regulations. This study was peer-reviewed with serious consideration given to the use of gene editing technology in human embryos for basic research by Institutional Review Board of the International Peace Maternity and Child Health Hospital (IPMCH). Upon completion of the review, the committee approved this novel research (research license number (GKLW) 2021-18) using gene editing technology under the conditions of strictly complying with current guidelines and policies in China. The approved study was monitored regularly by the IPMCH once the license was granted.

Before oocyte retrieval, all donors understood and signed the informed consent for donation of gametes for scientific research. All details of the proposed research project were clearly presented to donors, including the use of gene editing tools on gametes, embryos, and their derivatives (embryonic stem cell lines) to evaluate their safety and efficacy. Additionally, donors were informed that the donated gametes would not be used for other purposes, including but not limited to, conception for other people by assisted reproduction methods without informed consent of the study participants.

Materials and Methods

Gametes preparation and *in vitro* fertilization (IVF) in the clinic

Semen samples were collected by masturbation after 3−5 days of abstinence. Semen was kept at 37°C for 30 minutes for liquefaction, followed by an established density-gradient separation method. Briefly, after the second centrifugation, the pellet was re-suspended with 0.5 mL of G-IVF (Vitrolife) and incubated for standard swim-up for 30 min. The supernatant was used for insemination. For the acquisition of oocytes, cumulus-corona oocyte complexes (COCs) were isolated from the follicle fluid accurately and rapidly, and then cultured in G-IVF for 3 h. Next, each oocyte was inseminated in 4-well plates with approximately 100,000 motile spermatozoa. Approximately 18–20 h after fertilization, clinically abandoned three pro-nuclei (3PN) embryos were collected for experiments. In accordance with internationally accepted standards that developmental progression should not exceed 14 days, all human embryos used in this project were cultured *in vitro* for no more than 6 days, and then the subsequent genotyping analyses were performed.

Sperm cryopreservation and thawing

Semen samples were collected by masturbation from donors into sterile containers after 3–5 days of sexual abstinence and left to liquefy at 37°C. Semen samples were placed in 15 mL centrifuge tubes, diluted 1:1 with cryoprotectant in a slow drop-wise manner, and gently mixed to form a homogeneous solution. The homogeneous solutions of semen and cryoprotectant were equally aliquoted into 0.25 mL straws. Then, the straws were placed 4 cm (−170ºC to −180ºC) above the surface of liquid nitrogen for 15 min before being transferred into liquid nitrogen for preservation.

Straws containing homogeneous solution of semen and cryoprotectant were warmed in a water bath at 37°C for 1 min. The solution was then washed with 1 mL pre-warmed G-MOPS medium at least twice. The mixture was centrifuged at 360× g for 5 min to discard the supernatant, and then the pellet was resuspended in 100 μL G-MOPS medium, and stored at 37°C until it was used for intracytoplasmic sperm

injection (ICSI).

Generation of human 2PN embryos by ICSI

Immature metaphase I (MI) oocytes were collected from patients for IVF or ICSI treatment. All oocytes were retrieved with informed consent from the donor or patient. MI oocytes were cultured in a maturation medium (TCM199 + 10% FBS + 10 μg/mL sodium pyruvate + 10 μg/mL FSH + 5 μg/mL LH + 1 μg/mL E2+1 nM melatonin) until the first polar body appeared. Mature metaphase II (MII) oocytes were placed into a 50 μL micromanipulation droplet of HTF (modified human tubal fluid) with HEPES 10% buffer solution. The droplet was covered with tissue culture oil, and the dish was then mounted on the stage of an inverted microscope (Olympus IX73) equipped with a stage warmer and Narishige micromanipulators. Oocytes were fertilized by ICSI using frozen and thawed sperm. Fertilization was determined approximately 18 h after ICSI by noting the presence of two pronuclei and extrusion of the second polar body.

***In vitro* transcription of DdCBE mRNA**

The *ND6*-, *ND1*-, *ND4*, *ND5.1*, and *ATP8*-DdCBE plasmids were constructed in our lab, and the protein sequences encoding DdCBE used in the study are provided in the Supplementary sequence. The DdCBE plasmids were linearized, and the template was *in vitro* transcribed using the mMESSAGE mMACHINE T7 Ultra kit (Life Technologies) and purified using the MEGAclear kit (Life Technologies). The DdCBE mRNAs were individually re-suspended in RNase-free water, aliquoted, and stored at -80°C until use. Prior to microinjection, the DdCBE mRNAs were prepared by centrifuging for 10 min at 14,000 rpm at 4°C and then transferring the supernatant to a fresh 0.2 mL PCR tube for injection.

Microinjection of human 3PN or 2PN embryos with DdCBE

For 1-cell injection, the mixture of DdCBE-left mRNA (100 ng/μL) and DdCBE-right mRNA (100 ng/μL) was injected into the cytoplasm of zygotes 12-24 h after fertilization using a FemtoJet microinjector (Eppendorf) with constant flow

settings. For 2-cell, 4-cell, and 8-cell injection, the mixture of DdCBE-left mRNA (100 ng/μL) and DdCBE-right mRNA (100 ng/μL) was injected into every blastomere of the 2-cell, 4-cell, and 8-cell embryos about 30, 40, or 60 h after fertilization respectively. The injected embryos were cultured in drops of pre-equilibrated Global medium (LifeGlobal) under the conditions of 37°C, 6% CO_2, 5% O_2, and 89% N_2. Genotyping analysis was performed 2 days later.

Immunofluorescence staining

About 48 h after injection, embryos were labelled with MitoTracker Deep Red (Thermo Fisher) at a final concentration of 100 nM for 30 min in a 37 °C, 5% CO_2 incubator. Embryos were then fixed in 4% paraformaldehyde/PBS for 15 min at room temperature. Next, embryos were washed twice with PBS and permeabilized in PBS containing 0.1% Triton X-100 and 5% BSA for 2 h at room temperature. Embryos were then immunostained with anti-HA (Cell Signaling Technology, CST) and anti-FLAG (Sigma Aldrich), followed by Alexa Fluor-conjugated anti-rabbit and anti-mouse secondary antibodies (Abcam). The embryos were finally rinsed with 1× PBS 3 times and mounted onto slides with DAPI (Invitrogen) for nucleus staining. All images were captured using a confocal microscope (Olympus FV3000) and processed using Fiji software (NIH).

Mitochondrial base editing analysis

About 48 h after injection, individual embryos were placed into PCR tubes with 2.5 μL embryo lysis buffer (0.1% Tween-20, 0.1% Triton X-100, and 4 μg/mL proteinase K) and incubated at 56°C for 30 min, followed by heat inactivation at 95°C for 10 min. PCR amplification was performed using nested primer sets and Phanta Max Super-Fidelity DNA Polymerase (Vazyme). The first round PCR program was set as follows: 95°C for 30s, 60°C for 30s, and 72°C for 30s, with a final extension at 72°C for 5 min. The second round PCR was performed using 0.5 μL PCR product as the template and nested inner PCR primers, and carried out with the same program as the first round. The PCR product was analyzed by targeted deep sequencing to determine

the efficiency of base editing. PCR primers for genotyping analysis are included in Supplementary Table S1.

Targeted deep sequencing

Target sites were amplified by nested PCR from genomic DNA using Phanta Max Super-Fidelity DNA Polymerase (Vazyme). The paired-end sequencing of PCR amplicons was performed with GENEWIZ Co., Ltd using the NovaSeq 6000 platform. The sequencing data were subsequently demultiplexed using fastq-multx (v1.4.1) with the PCR primers. Next, sequence alignment was performed between the demultiplexed sequencing data with each of the on- and off-target sites using CRISPResso2 (v2.0.32), and mapping statistics was generated using in-house scripts with Perl (v5.26.2) and R (v4.1.0).

Mitochondrial genome-wide off-target analysis

About 50-60 h after ICSI, half of the 8-cell embryos were injected with a mixture of *GFP* and DdCBE mRNA, and the other half were not injected, as the control group. Subsequently, zonae pellucidae from 16-cell stage embryos were removed by brief exposure to acidic Tyrode solution (Sigma Aldrich). Zona-free embryos were briefly (30s) exposed to the 0.05% Trypsin-EDTA solution (Gibco) before manual disaggregation into single blastomeres with a small bore pipette. Individual GFP^+ and GFP^- blastomeres were transferred into 0.2 mL PCR tubes containing 4 μL PBS and placed into a freezer at -80°C until further use. Whole genome amplification of the blastomeres was then performed using the REPLI-g Single Cell Kit (Qiagen). Briefly, samples frozen at -80°C were thawed and transferred into PCR tubes containing reconstituted buffer D2 (7 μL), and then incubated at 65°C for 10 min before the addition of stop solution (3.5 μL) and master mix (40 μL), followed by incubation at 30°C for 8 h. The DNA preparation was diluted with ddH_2O (1:30), and 1 μL of the diluted DNA was used for PCR analysis and targeted deep sequencing.

The whole mitochondrial genome sequencing was performed on the Illumina NovaSeq 6000 platform. Trimmomatic (v0.39) was used to trim the low quality reads

and adapter sequences from the FASTQ files. Qualified reads were mapped to the human mitochondrial reference genome (hg19) by BWA (v0.7.12) with mem –M, and Picard-tools (v2.3.0) was used to reorder, sort, add read groups, and mark duplicates of the aligned BAM files. Then, Strelka (v2.7.1), Lofreq (v2.1.2), and Mutect2 (v4.1.5) were run on the aligned BAM files of GFP^+ cells for *de novo* single nucleotide variants (SNVs) detection, with GFP^- cells in the same embryo as control. For example, if the WT allele is G in a certain coordinate, the GFP^+ cells carries A, and the GFP^- cells carries G, then the mutant A will be called as a *de novo* mutation. While, if GFP^- cells carries A, the mutant could not be identified. Only variants identified by all three algorithms were included in the following analysis. To strictly control the quality of the variants, we removed variants that overlapped with UCSC repeat regions or that were reported in the dbSNP151 database.

Statistical analysis

All statistical values are presented as means ± SEM. Differences between datasets were considered to be significant at *P* value less than 0.05. All statistical tests were conducted with the unpaired student's *t*-test (two-tailed), unless otherwise stated.

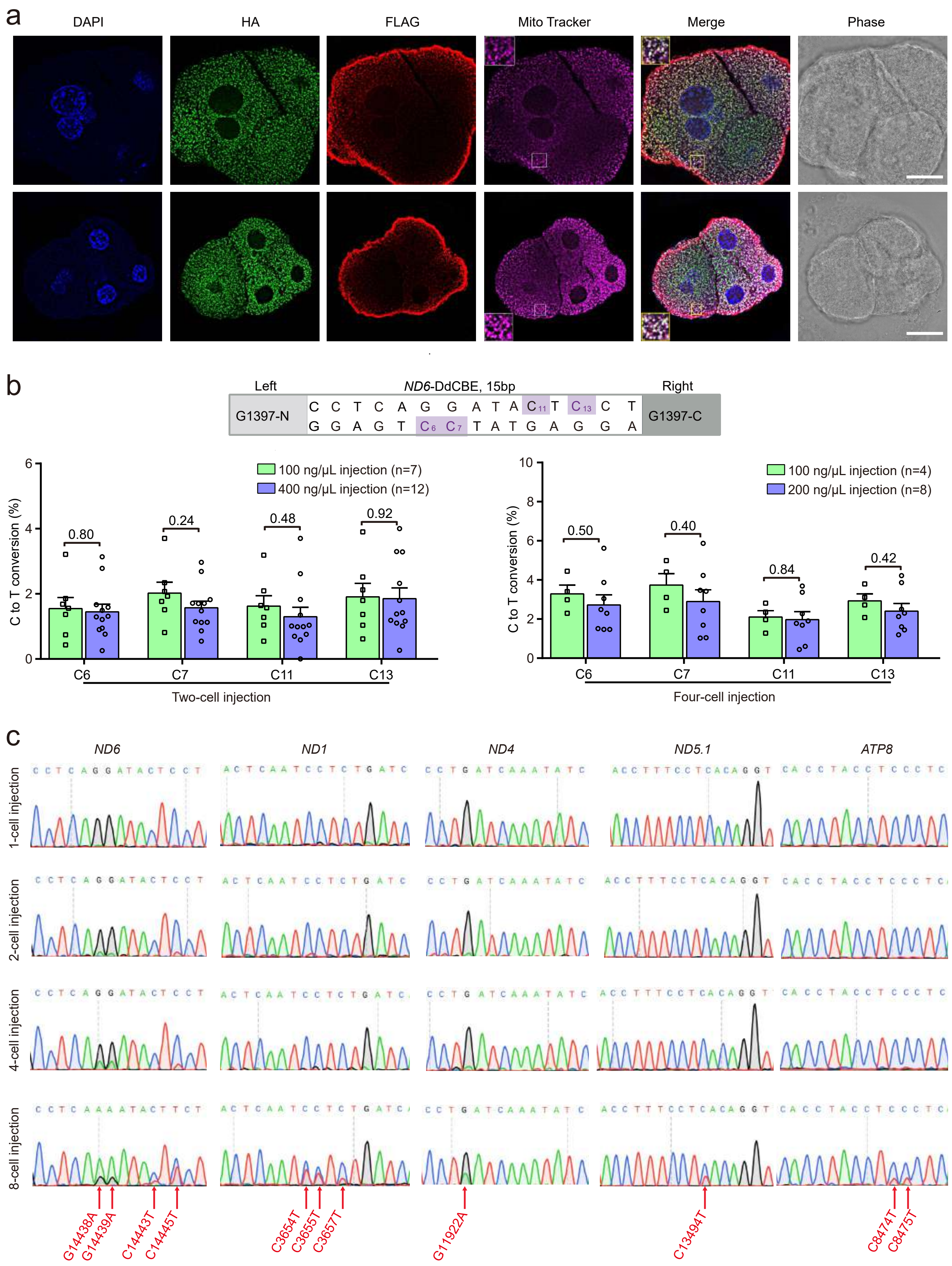

a
DAPI
HA
FLAG
Mito Tracker
Merge
Phase
b
Left
ND6-DdCBE, 15bp
Right
G1397-N
C C T C A G G A T A C11 T C13 C T
G G A G T C6 C7 T A T G A G G A
G1397-C
100 ng/μL injection (n=7)
400 ng/μL injection (n=12)
C to T conversion (%)
0.80
0.24
0.48
0.92
C6
C7
C11
C13
Two-cell injection
100 ng/μL injection (n=4)
200 ng/μL injection (n=8)
0.50
0.40
0.84
0.42
Four-cell injection
c
ND6
ND1
ND4
ND5.1
ATP8
1-cell injection
2-cell injection
4-cell injection
8-cell injection
G14438A
G14439A
C14443T
C14445T
C3654T
C3655T
C3657T
G11922A
C13494T
C8474T
C8475T

Supplementary Fig. S1. DdCBE expression, cellular localization and editing efficiency in human zygote and cleaving 3PN embryos. a. Fluorescence image of HA- and FLAG-tagged halves of UGI-TALE-split DddAtox and TALE-split DddAtox-UGI pairs in human embryos 24-48h after injecting the corresponding mRNAs. Scale bars, 25μm. Mitochondrial localization was followed using MitoTracker (magenta) **b.** DdCBE concentration analysis for two-cell and four-cell injection. Data are presented as means ± SEM. *P* values were evaluated with the unpaired student's *t*-test (two-tailed). **c.** Sanger sequencing results of mtDNA editing with *ND6*-, *ND1*-, *ND4*-, *ND5.1*-, and *ATP8*-DdCBE.

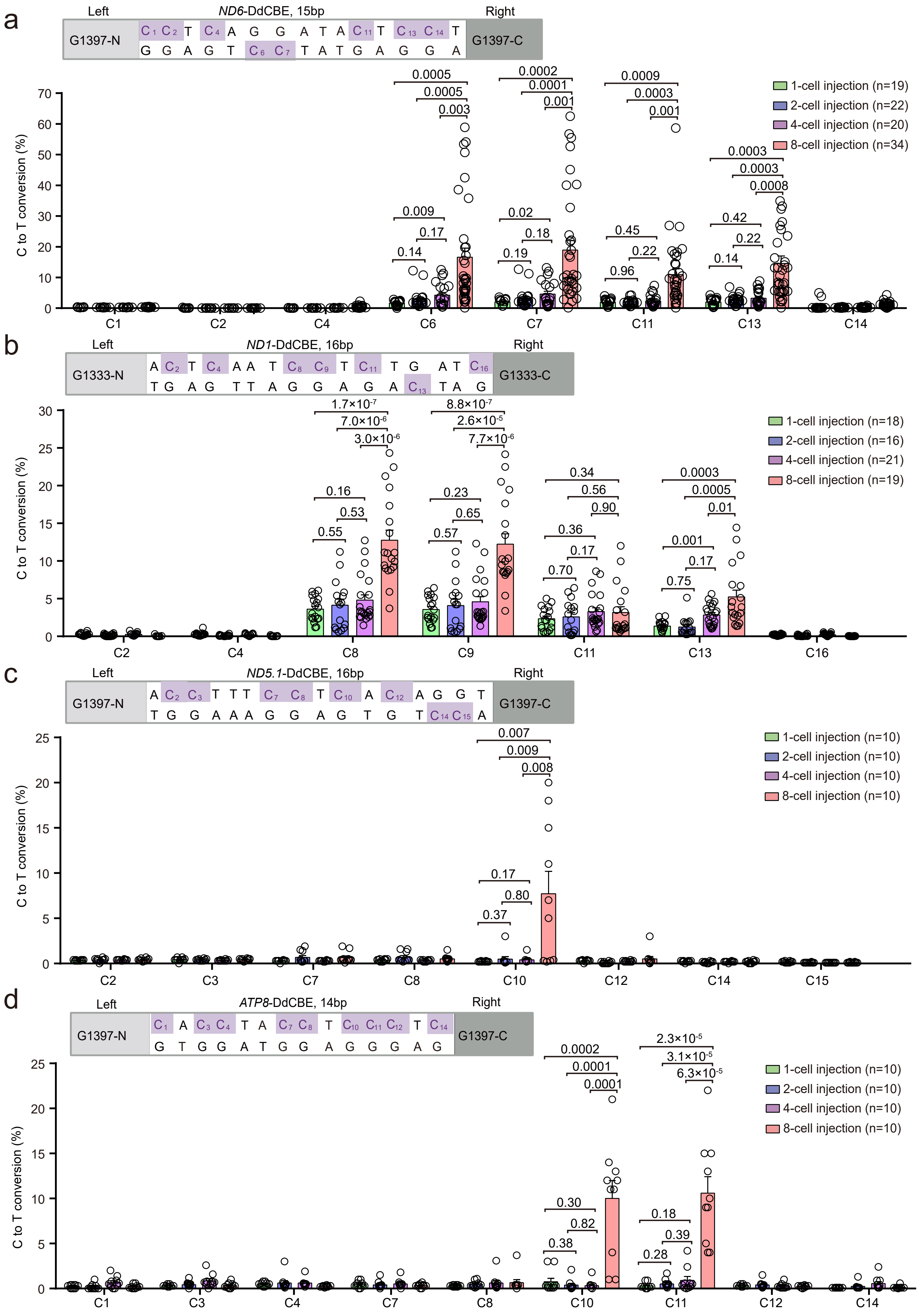
a
Left
ND6-DdCBE, 15bp
Right
G1397-N
C1 C2 T C4 A G G A T A C11 T C13 C14 T
G G A G T C6 C7 T A T G A G G A
G1397-C
C to T conversion (%)
1-cell injection (n=19)
2-cell injection (n=22)
4-cell injection (n=20)
8-cell injection (n=34)
0.0005
0.0005
0.003
0.009
0.17
0.14
0.0002
0.0001
0.001
0.02
0.18
0.19
0.0009
0.0003
0.001
0.45
0.22
0.96
0.0003
0.0003
0.0008
0.42
0.22
0.14
C1 C2 C4 C6 C7 C11 C13 C14
b
Left
ND1-DdCBE, 16bp
Right
G1333-N
A C2 T C4 A A T C8 C9 T C11 T G A T C16
T G A G T T A G G A G A C13 T A G
G1333-C
C to T conversion (%)
1-cell injection (n=18)
2-cell injection (n=16)
4-cell injection (n=21)
8-cell injection (n=19)
1.7×10-7
7.0×10-6
3.0×10-6
0.16
0.53
0.55
8.8×10-7
2.6×10-5
7.7×10-6
0.23
0.65
0.57
0.34
0.56
0.90
0.36
0.17
0.70
0.0003
0.0005
0.01
0.001
0.17
0.75
C2 C4 C8 C9 C11 C13 C16
c
Left
ND5.1-DdCBE, 16bp
Right
G1397-N
A C2 C3 T T T C7 C8 T C10 A C12 A G G T
T G G A A A G G A G T G T C14 C15 A
G1397-C
C to T conversion (%)
1-cell injection (n=10)
2-cell injection (n=10)
4-cell injection (n=10)
8-cell injection (n=10)
0.007
0.009
0.008
0.17
0.80
0.37
C2 C3 C7 C8 C10 C12 C14 C15
d
Left
ATP8-DdCBE, 14bp
Right
G1397-N
C1 A C3 C4 T A C7 C8 T C10 C11 C12 T C14
G T G G A T G G A G G G A G
G1397-C
C to T conversion (%)
1-cell injection (n=10)
2-cell injection (n=10)
4-cell injection (n=10)
8-cell injection (n=10)
0.0002
0.0001
0.0001
0.30
0.82
0.38
2.3×10-5
3.1×10-5
6.3×10-5
0.18
0.39
0.28
C1 C3 C4 C7 C8 C10 C11 C12 C14

Supplementary Fig. S2. Base editing efficiency of DdCBE in human 1-cell, 2-cell, 4-cell, 8-cell embryos of 3PN. a–d. Base editing efficiency of *ND6*-DdCBE (**a**), *ND1*-DdCBE (**b**), *ND5.1*-DdCBE (**c**), *ATP8*-DdCBE (**d**) in human 1-cell, 2-cell, 4-cell, 8-cell embryos of 3PN. Data are presented as means ± SEM. *P* values were evaluated with the unpaired student's *t*-test (two-tailed).

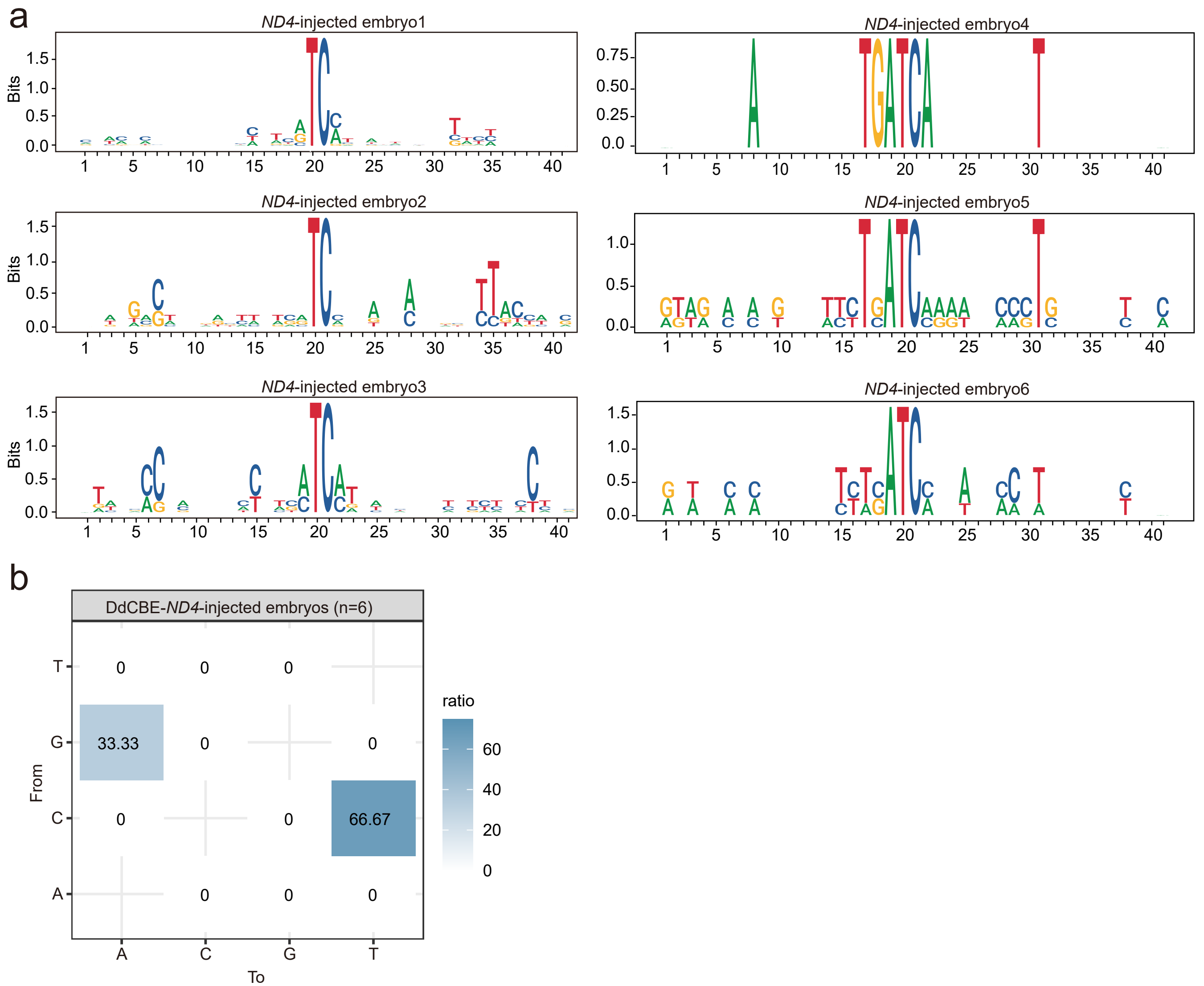
a
ND4-injected embryo1
ND4-injected embryo2
ND4-injected embryo3
ND4-injected embryo4
ND4-injected embryo5
ND4-injected embryo6
Bits
b
DdCBE-ND4-injected embryos (n=6)
From
To
T
G
C
A
33.33
66.67
ratio
60
40
20
0

Supplementary Fig. S3. Off-target profiling results for *ND4*-DdCBE. a. Sequence logos generated from sequences with off-target C·G to T·A conversions on mitochondrial DNA in six *ND4*-DdCBE-injected embryos. Bits reflect sequence conservation at a given position. **b.** Heatmap showing ratios of different base-conversion types induced by *ND4*-DdCBE at off-target loci.

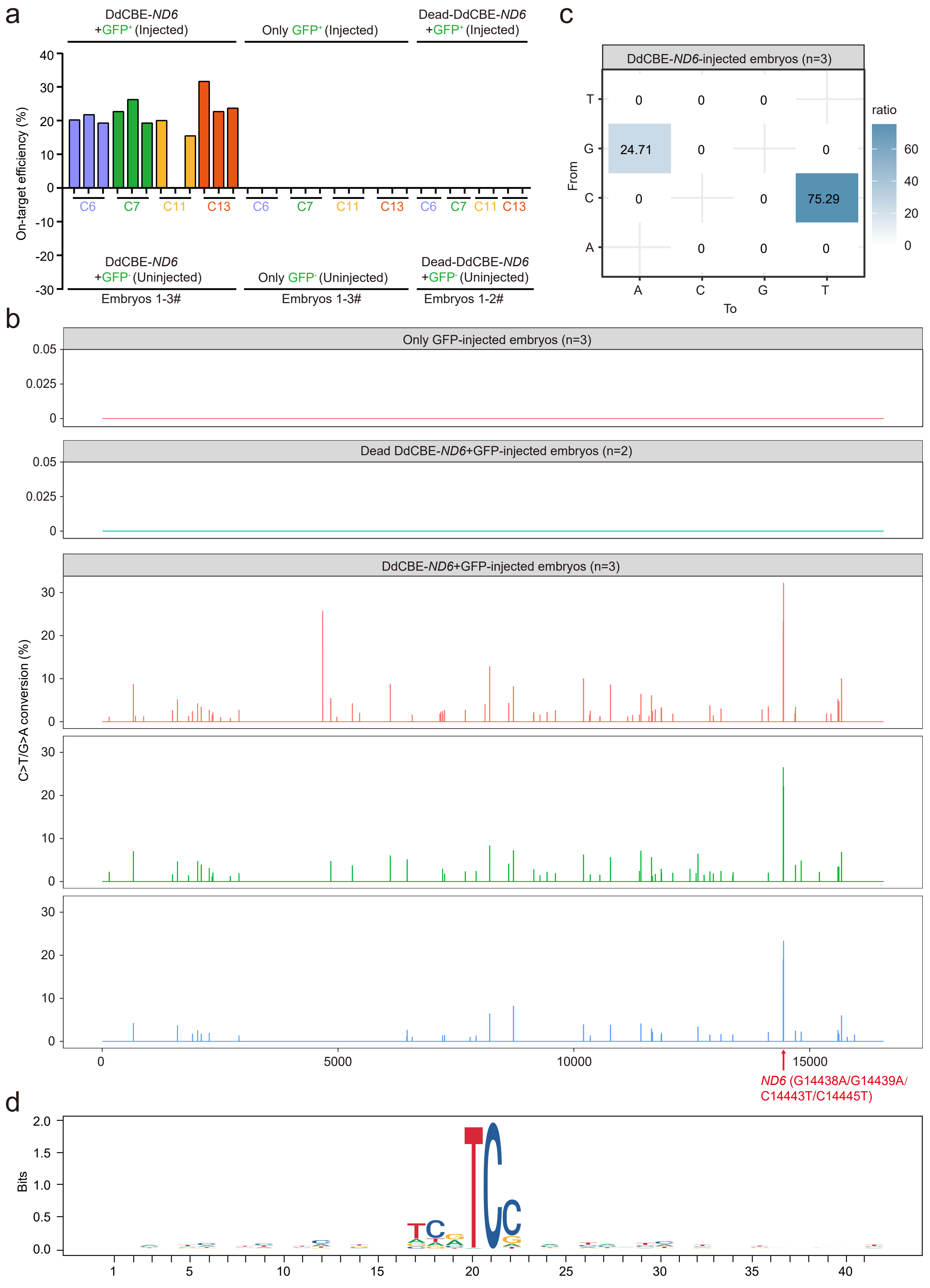
a
DdCBE-ND6 +GFP+ (Injected)
Only GFP+ (Injected)
Dead-DdCBE-ND6 +GFP+ (Injected)
On-target efficiency (%)
C6 C7 C11 C13 C6 C7 C11 C13 C6 C7 C11 C13
DdCBE-ND6 +GFP- (Uninjected)
Only GFP- (Uninjected)
Dead-DdCBE-ND6 +GFP- (Uninjected)
Embryos 1-3#
Embryos 1-3#
Embryos 1-2#
c
DdCBE-ND6-injected embryos (n=3)
From
To
ratio
24.71
75.29
b
Only GFP-injected embryos (n=3)
Dead DdCBE-ND6+GFP-injected embryos (n=2)
DdCBE-ND6+GFP-injected embryos (n=3)
C>T/G>A conversion (%)
ND6 (G14438A/G14439A/ C14443T/C14445T)
d
Bits

Supplementary Fig. S4. Off-target profiling results for *ND6*-DdCBE. a. On-target efficiency of *ND6*-DdCBE in human 2PN embryos. Embryos injected with *ND6*-Dead-DdCBE + *GFP* or only *GFP* mRNA showed no base editing on the *ND6* site. **b.** Mitochondrial genome-wide off-target loci identified from human 2PN embryos injected with *ND6*-DdCBE. No off-target SNV was identified in control embryos injected with *ND6*-Dead-DdCBE + *GFP* or *GFP* mRNA only whereas tens of SNVs were identified in embryos injected with both *GFP* and *ND6*-DdCBE mRNA possibly due to off-target editing. **c.** Heatmap showing ratios of different base-conversion types induced by *ND6*-DdCBE at off-target loci. **d.** Sequence logos generated from sequences with off-target C·G to T·A conversions by *ND6*-DdCBE in mitochondrial genome. Bits reflect sequence conservation at a given position.

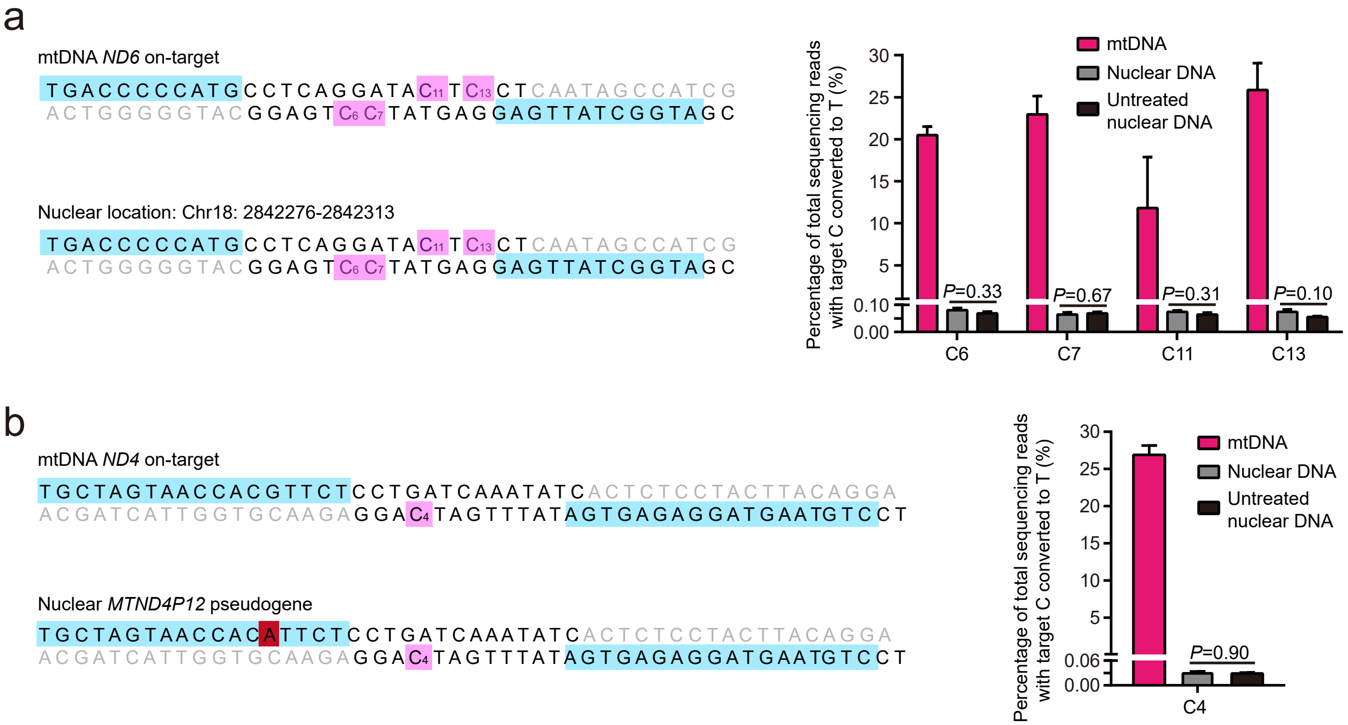
a
mtDNA ND6 on-target
TGACCCCCATGCCTCAGGATAC11TC13CTCAATAGCCATCG
ACTGGGGGTACGGAGTC6C7TATGAGGAGTTATCGGTAGC
Nuclear location: Chr18: 2842276-2842313
TGACCCCCATGCCTCAGGATAC11TC13CTCAATAGCCATCG
ACTGGGGGTACGGAGTC6C7TATGAGGAGTTATCGGTAGC
Percentage of total sequencing reads with target C converted to T (%)
mtDNA
Nuclear DNA
Untreated nuclear DNA
P=0.33
P=0.67
P=0.31
P=0.10
C6
C7
C11
C13
b
mtDNA ND4 on-target
TGCTAGTAACCACGTTCTCCTGATCAAATATCACTCTCCTACTTACAGGA
ACGATCATTGGTGCAAGAGGAC4TAGTTTATAGTGAGAGGATGAATGTCCT
Nuclear MTND4P12 pseudogene
TGCTAGTAACCACATTCTCCTGATCAAATATCACTCTCCTACTTACAGGA
ACGATCATTGGTGCAAGAGGAC4TAGTTTATAGTGAGAGGATGAATGTCCT
Percentage of total sequencing reads with target C converted to T (%)
mtDNA
Nuclear DNA
Untreated nuclear DNA
P=0.90
C4

Supplementary Fig. S5. Off-target analysis of DdCBEs on nuclear DNA in human embryos. a, b. Left: the on-target editing sites on mtDNA and the corresponding nuclear DNA sequence with the greatest homology are shown for *ND6*-DdCBE (**a**) and *ND4*-DdCBE (**b**). TALE binding sites begin at N0 and are shown in blue. Target cytosines are in purple. Nucleotide mismatches between the mtDNA and nuclear pseudogene are in red. Right: editing efficiencies are measured by targeted deep sequencing (see Supplemenatry Table S1 for primer sequences). Data are presented as means ± SEM. *P* values were evaluated with the unpaired student's *t*-test (two-tailed).

Supplementary Table S1. List of PCR primers used for Sanger sequencing or targeted deep sequencing

Targeting sites	Primer name	Sequence (5'-3')
ND6	Human mito-*ND6* Genotyping/HTS OF	ttgattgttagcggtgtggtc
	Human mito-*ND6* Genotyping/HTS OR	atactctttcacccacagcacc
	Human mito-*ND6* Genotyping IF	tgttagcggtgtggtcggg
	Human mito-*ND6* Genotyping IR	ctctttcacccacagcaccaatc
	Human mito-*ND6* HTS IF	gtNNNNNNNNNNcggtgtggtcgggtgtgttatt
	Human mito-*ND6* HTS IR	caatcctacctccatcgctaac
	Human mito-*ND6* nuclear HTS OF	ctgaagagggcaagtgttctttg
	Human mito-*ND6* nuclear HTS OR	gaatgccaggatatgggcaataca
	Human mito-*ND6* nuclear HTS IF	gaattcNNNNNNgaagtctggagactcactga
	Human mito-*ND6* nuclear HTS IR	ctcctgagatcaagcaatcc
ND1	Human mito-*ND1* Genotyping/HTS OF	atggcattcctaatgcttaccga
	Human mito-*ND1* Genotyping/HTS OR	agttcaggggagagtgcgt
	Human mito-*ND1* Genotyping IF	gcccctacgggctactacaac
	Human mito-*ND1* Genotyping IR	cctgcggcgtattcgatgt
	Human mito-*ND1* HTS IF	gtNNNNNNNNNNctctcaccatcgctcttctact
	Human mito-*ND1* HTS IR	gaatgatggctagggtgacttc
ND4	Human mito-*ND4* Genotyping/HTS OF	gccattctcatccaaacc
	Human mito-*ND4* Genotyping/HTS OR	ggttgagggataggaggag
	Human mito-*ND4* Genotyping IF	cataatcgcccacgggctta
	Human mito-*ND4* Genotyping IR	taggtgtatgaacatgaggg
	Human mito-*ND4* HTS IF	gtNNNNNNNNNNgatgacttctagcaagcctcg
	Human mito-*ND4* HTS IR	gttaatgtggtgggtgagtgagc
	Human mito-*ND4* nuclear HTS OF	ctaattctctttgaggagcatggttag
	Human mito-*ND4* nuclear HTS OR	tatcacttccagccacctatttcc
	Human mito-*ND4* nuclear HTS IF	ctatatttacaggaggaaaacccgg
	Human mito-*ND4* nuclear HTS IR	gaattcNNNNNNgacttctagcaagcctcactaatc
ND5.1	Human mito-*ND5.1* Genotyping/HTS OF	tcggcatcaaccaaccacac
	Human mito-*ND5.1* Genotyping/HTS OR	gaagtcctaggaaagtgacagcg
	Human mito-*ND5.1* Genotyping IF	cacatctgtacccacgcct
	Human mito-*ND5.1* Genotyping IR	gggggaaatgttgttagtaatgaga
	Human mito-*ND5.1* HTS IF	NNNNNNNNNNtccgggtccatcatccacaa
	Human mito-*ND5.1* HTS IR	cgagtgctataggcgcttgt
ATP8	Human mito-*ATP8* Genotyping/HTS OF	cctatagcacccccctctaccc
	Human mito-*ATP8* Genotyping/HTS OR	aatgatcagtactgcggcgg
	Human mito-*ATP8* Genotyping IF	ttacagtgaaatgccccaac
	Human mito-*ATP8* Genotyping IR	tgggggcaatgaatgaagcg
	Human mito-*ATP8* HTS IF	NNNNNNNNNNctaaatactaccgtatggcc
	Human mito-*ATP8* HTS IR	gatcagtactgcggcgggta

Supplementary sequence:

DdCBE domains are annotated as: yellow for MTS, italics for linker, cyan for HA or FLAG tag, purple for N&C-terminal domain, underlined for RVD, red for half of DddAtox, green for UGI.

ND6-DdCBE: Left-SOD2 MTS-3×HA-mitoTALE-G1397-DddA$_{tox}$-N-1×UGI
MALSRAVCGTSRQLAPVLGYLGSRQKHSLPDYPYDVPDYAGYPYDVPDYAG
YPYDVPDYAMDIADLRTLGYSQQQQEKIKPKVRSTVAQHHEALVGHGFTHA
HIVALSQHPAALGTVAVKYQDMIAALPEATHEAIVGVGKQWSGARALEALL
TVAGELRGPPLQLDTGQLLKIAKRGGVTAVEAVHAWRNALTGAPLNLTPQQ
VVAIASNNGGKQALETVQRLLPVLCQAHGLTPEQVVAIASNIGGKQALETVQ
ALLPVLCQAHGLTPEQVVAIASHDGGKQALETVQRLLPVLCQAHGLTPEQVV
AIASHDGGKQALETVQRLLPVLCQAHGLTPEQVVAIASHDGGKQALETVQRL
LPVLCQAHGLTPEQVVAIASHDGGKQALETVQRLLPVLCQAHGLTPEQVVAI
ASHDGGKQALETVQRLLPVLCQAHGLTPEQVVAIASNIGGKQALETVQALLP
VLCQAHGLTPQQVVAIASNGGGKQALETVQRLLPVLCQAHGLTPQQVVAIAS
NNGGRPALESIVAQLSRPDPALAALTNDHLVALACLGGRPALDAVKKGLG*GS*
GSYALGPYQISAPQLPAYNGQTVGTFYYVNDAGGLESKVFSSGGPTPYPNYA
NAGHVEGQSALFMRDNGISEGLVFHNNPEGTCGFCVNMTETLLPENAKMTV
VPPEG*SGGS*TNLSDIIEKETGKQLVIQESILMLPEEVEEVIGNKPESDILVHTAY
DESTDENVMLLTSDAPEYKPWALVIQDSNGENKIKML*

ND6-DdCBE: Right-COX8A MTS-3×FLAG-mitoTALE-G1397-DddA$_{tox}$-C-1×UGI
MASVLTPLLLRGLTGSARRLPVPRAKIHSLDYKDHDGDYKDHDIDYKDDDD
KMDIADLRTLGYSQQQQEKIKPKVRSTVAQHHEALVGHGFTHAHIVALSQHP
AALGTVAVKYQDMIAALPEATHEAIVGVGKRGAGARALEALLTVAGELRGP
PLQLDTGQLLKIAKRGGVTAVEAVHAWRNALTGAPLNLTPQQVVAIASNNG
GKQALETVQRLLPVLCQAHGLTPEQVVAIASNIGGKQALETVQALLPVLCQA
HGLTPQQVVAIASNGGGKQALETVQRLLPVLCQAHGLTPQQVVAIASNNGG
KQALETVQRLLPVLCQAHGLTPQQVVAIASNNGGKQALETVQRLLPVLCQA
HGLTPEQVVAIASHDGGKQALETVQRLLPVLCQAHGLTPQQVVAIASNGGG
KQALETVQRLLPVLCQAHGLTPEQVVAIASNIGGKQALETVQALLPVLCQAH
GLTPQQVVAIASNGGGKQALETVQRLLPVLCQAHGLTPQQVVAIASNGGGK
QALETVQRLLPVLCQAHGLTPQQVVAIASNNGGRPALESIVAQLSRPDPALA
ALTNDHLVALACLGGRPALDAVKKGLG*GS*AIPVKRGATGETKVFTGNSNSPK
SPTKGGC*SGGS*TNLSDIIEKETGKQLVIQESILMLPEEVEEVIGNKPESDILVHT
AYDESTDENVMLLTSDAPEYKPWALVIQDSNGENKIKML*

ND1-DdCBE: Left-SOD2 MTS-3×HA-mitoTALE-G1333-DddA$_{tox}$-N-1×UGI
MALSRAVCGTSRQLAPVLGYLGSRQKHSLPDYPYDVPDYAGYPYDVPDYAG
YPYDVPDYAMDIADLRTLGYSQQQQEKIKPKVRSTVAQHHEALVGHGFTHA

HIVALSQHPAALGTVAVKYQDMIAALPEATHEAIVGVGKQWSGARALEALL
TVAGELRGPPLQLDTGQLLKIAKRGGVTAVEAVHAWRNALTGAPLNLTPEQ
VVAIASHDGGKQALETVQALLPVLCQAHGLTPQQVVAIASNGGGKQALETV
QRLLPVLCQAHGLTPQQVVAIASNIGGKQALETVQRLLPVLCQAHGLTPEQV
VAIASNNGGKQALETVQALLPVLCQAHGLTPEQVVAIASHDGGKQALETVQ
ALLPVLCQAHGLTPEQVVAIASHDGGKQALETVQALLPVLCQAHGLTPEQVV
AIASNGGGKQALETVQRLLPVLCQAHGLTPEQVVAIASNIGGKQALETVQAL
LPVLCQAHGLTPEQVVAIASNNGGKQALETVQRLLPVLCQAHGLTPEQVVAI
ASHDGGKQALETVQALLPVLCQAHGLTPEQVVAIASHDGGKQALETVQALL
PVLCQAHGLTPEQVVAIASNNGGKQALETVQALLPVLCQAHGLTPEQVVAIA
SNGGGKQALETVQRLLPVLCQAHGLTPQQVVAIASNGGGKQALETVQRLLP
VLCQAHGLTPQQVVAIASNGGGRPALESIVAQLSRPDPALAALTNDHLVALA
CLGGRPALDAVKKGLG*GS*GSYALGPYQISAPQLPAYNGQTVGTFYYVNDAG
GLESKVFSSGG*SGGS*TNLSDIIEKETGKQLVIQESILMLPEEVEEVIGNKPESDIL
VHTAYDESTDENVMLLTSDAPEYKPWALVIQDSNGENKIKML*

ND1-DdCBE: Right-COX8A MTS-3×FLAG-mitoTALE-G1333-DddA$_{tox}$-C-1×UGI
MASVLTPLLLRGLTGSARRLPVPRAKIHSLDYKDHDGDYKDHDIDYKDDDD
KMDIADLRTLGYSQQQQEKIKPKVRSTVAQHHEALVGHGFTHAHIVALSQHP
AALGTVAVKYQDMIAALPEATHEAIVGVGKQWSGARALEALLTVAGELRGP
PLQLDTGQLLKIAKRGGVTAVEAVHAWRNALTGAPLNLTPEQVVAIASNNG
GKQALETVQALLPVLCQAHGLTPQQVVAIASNIGGKQALETVQRLLPVLCQA
HGLTPQQVVAIASNNGGKQALETVQRLLPVLCQAHGLTPEQVVAIASNGGG
KQALETVQALLPVLCQAHGLTPEQVVAIASNGGGKQALETVQALLPVLCQA
HGLTPEQVVAIASNGGGKQALETVQALLPVLCQAHGLTPEQVVAIASNNGGK
QALETVQRLLPVLCQAHGLTPEQVVAIASNIGGKQALETVQALLPVLCQAHG
LTPEQVVAIASNGGGKQALETVQRLLPVLCQAHGLTPEQVVAIASNNGGKQA
LETVQALLPVLCQAHGLTPEQVVAIASHDGGKQALETVQALLPVLCQAHGLT
PEQVVAIASNGGGKQALETVQALLPVLCQAHGLTPEQVVAIASHDGGKQALE
TVQRLLPVLCQAHGLTPQQVVAIASNIGGKQALETVQRLLPVLCQAHGLTPE
QVVAIASHDGGKQALETVQALLPVLCQAHGLTPQQVVAIASHDGGKQALET
VQRLLPVLCQAHGLTPQQVVAIASHDGGKQALETVQRLLPVLCQAHGLTPQ
QVVAIASNGGGRPALESIVAQLSRPDPALAALTNDHLVALACLGGRPALDAV
KKGLG*GS*PTPYPNYANAGHVEGQSALFMRDNGISEGLVFHNNPEGTCGFCVN
MTETLLPENAKMTVVPPEGAIPVKRGATGETKVFTGNSNSPKSPTKGGC*SGGS*
TNLSDIIEKETGKQLVIQESILMLPEEVEEVIGNKPESDILVHTAYDESTDENVM
LLTSDAPEYKPWALVIQDSNGENKIKML*

ND4-DdCBE: Left-SOD2 MTS-3×HA-mitoTALE-G1397-DddA$_{tox}$-C-1×UGI
MALSRAVCGTSRQLAPVLGYLGSRQKHSLPDYPYDVPDYAGYPYDVPDYAG
YPYDVPDYAMDIADLRTLGYSQQQQEKIKPKVRSTVAQHHEALVGHGFTHA
HIVALSQHPAALGTVAVKYQDMIAALPEATHEAIVGVGKQWSGARALEALL

TVAGELRGPPLQLDTGQLLKIAKRGGVTAVEAVHAWRNALTGAPLNLTPEQ
VVAIASNNGGKQALETVQALLPVLCQAHGLTPQQVVAIASHDGGKQALETV
QRLLPVLCQAHGLTPQQVVAIASNGGGKQALETVQRLLPVLCQAHGLTPEQV
VAIASNIGGKQALETVQALLPVLCQAHGLTPEQVVAIASNNGGKQALETVQA
LLPVLCQAHGLTPEQVVAIASNGGGKQALETVQALLPVLCQAHGLTPEQVVA
IASNIGGKQALETVQRLLPVLCQAHGLTPEQVVAIASNIGGKQALETVQALLP
VLCQAHGLTPEQVVAIASHDGGKQALETVQRLLPVLCQAHGLTPEQVVAIAS
HDGGKQALETVQALLPVLCQAHGLTPEQVVAIASNIGGKQALETVQALLPVL
CQAHGLTPEQVVAIASHDGGKQALETVQALLPVLCQAHGLTPEQVVAIASNN
GGKQALETVQRLLPVLCQAHGLTPQQVVAIASNGGGKQALETVQRLLPVLC
QAHGLTPEQVVAIASNGGGKQALETVQALLPVLCQAHGLTPEQVVAIASHDG
GKQALETVQALLPVLCQAHGLTPQQVVAIASNGGGRPALESIVAQLSRPDPA
LAALTNDHLVALACLGGRPALDAVKKGLG*GS*AIPVKRGATGETKVFTGNSN
SPKSPTKGGC*SGGS*TNLSDIIEKETGKQLVIQESILMLPEEVEEVIGNKPESDILV
HTAYDESTDENVMLLTSDAPEYKPWALVIQDSNGENKIKML*

ND4-DdCBE: Right-COX8A MTS-3×FLAG-mitoTALE-G1397-DddA$_{tox}$-N-1×UGI
MASVLTPLLLRGLTGSARRLPVPRAKIHSLDYKDHDGDYKDHDIDYKDDDD
KMDIADLRTLGYSQQQQEKIKPKVRSTVAQHHEALVGHGFTHAHIVALSQHP
AALGTVAVKYQDMIAALPEATHEAIVGVGKQWSGARALEALLTVAGELRGP
PLQLDTGQLLKIAKRGGVTAVEAVHAWRNALTGAPLNLTPEQVVAIASHDG
GKQALETVQALLPVLCQAHGLTPQQVVAIASHDGGKQALETVQRLLPVLCQ
AHGLTPQQVVAIASNGGGKQALETVQRLLPVLCQAHGLTPEQVVAIASNNG
GKQALETVQALLPVLCQAHGLTPEQVVAIASNGGGKQALETVQALLPVLCQ
AHGLTPEQVVAIASNIGGKQALETVQALLPVLCQAHGLTPEQVVAIASNIGGK
QALETVQRLLPVLCQAHGLTPEQVVAIASNNGGKQALETVQALLPVLCQAH
GLTPEQVVAIASNGGGKQALETVQRLLPVLCQAHGLTPEQVVAIASNIGGKQ
ALETVQRLLPVLCQAHGLTPEQVVAIASNNGGKQALETVQALLPVLCQAHGL
TPEQVVAIASNNGGKQALETVQALLPVLCQAHGLTPEQVVAIASNIGGKQAL
ETVQALLPVLCQAHGLTPEQVVAIASNNGGKQALETVQRLLPVLCQAHGLTP
QQVVAIASNIGGKQALETVQRLLPVLCQAHGLTPQQVVAIASNNGGKQALET
VQRLLPVLCQAHGLTPQQVVAIASNGGGRPALESIVAQLSRPDPALAALTND
HLVALACLGGRPALDAVKKGLG*GS*GSYALGPYQISAPQLPAYNGQTVGTFY
YVNDAGGLESKVFSSGGPTPYPNYANAGHVEGQSALFMRDNGISEGLVFHN
NPEGTCGFCVNMTETLLPENAKMTVVPPEG*SGGS*TNLSDIIEKETGKQLVIQES
ILMLPEEVEEVIGNKPESDILVHTAYDESTDENVMLLTSDAPEYKPWALVIQD
SNGENKIKML*

ND5.1-DdCBE: Left-SOD2 MTS-3×HA-mitoTALE-G1397-DddA$_{tox}$-N-1×UGI
MALSRAVCGTSRQLAPVLGYLGSRQKHSLPDYPYDVPDYAGYPYDVPDYAG
YPYDVPDYAMDIADLRTLGYSQQQQEKIKPKVRSTVAQHHEALVGHGFTHA
HIVALSQHPAALGTVAVKYQDMIAALPEATHEAIVGVGKQWSGARALEALL

TVAGELRGPPLQLDTGQLLKIAKRGGVTAVEAVHAWRNALTGAPLNLTPDQ
VVAIASNIGGKQALETVQRLLPVLCQAHGLTPDQVVAIASNNGGKQALETVQ
RLLPVLCQAHGLTPDQVVAIASHDGGKQALETVQRLLPVLCQAHGLTPAQV
VAIASNIGGKQALETVQRLLPVLCQAHGLTPDQVVAIASNGGGKQALETVQR
LLPVLCQAHGLTPAQVVAIASNGGGKQALETVQRLLPVLCQAHGLTPDQVV
AIASNIGGKQALETVQRLLPVLCQAHGLTPDQVVAIASNNGGKQALETVQRL
LPVLCQAHGLTPDQVVAIASHDGGKQALETVQRLLPVLCQAHGLTPAQVVAI
ASNIGGKQALETVQRLLPVLCQAHGLTPDQVVAIASNNGGKQALETVQRLLP
VLCQAHGLTPDQVVAIASNNGGKQALETVQRLLPVLCQAHGLTPDQVVAIAS
NIGGKQALETVQRLLPVLCQAHGLTPDQVVAIASNIGGKQALETVQRLLPVL
CQAHGLTPDQVVAIASNGGGRPALESIVAQLSRPDPALAALTNDHLVALACL
GGRPALDAVKKGLG*GS*GSYALGPYQISAPQLPAYNGQTVGTFYYVNDAGGL
ESKVFSSGGPTPYPNYANAGHVEGQSALFMRDNGISEGLVFHNNPEGTCGFC
VNMTETLLPENAKMTVVPPEG*SGGS*TNLSDIIEKETGKQLVIQESILMLPEEVE
EVIGNKPESDILVHTAYDESTDENVMLLTSDAPEYKPWALVIQDSNGENKIK
ML*

ND5.1-DdCBE: Right-COX8A MTS-3×FLAG-mitoTALE-G1397-DddA$_{tox}$-C-1×UGI
MASVLTPLLLRGLTGSARRLPVPRAKIHSLDYKDHDGDYKDHDIDYKDDDD
KMDIADLRTLGYSQQQQEKIKPKVRSTVAQHHEALVGHGFTHAHIVALSQHP
AALGTVAVKYQDMIAALPEATHEAIVGVGKQWSGARALEALLTVAGELRGP
PLQLDTGQLLKIAKRGGVTAVEAVHAWRNALTGAPLNLTPDQVVAIASHDG
GKQALETVQRLLPVLCQAHGLTPAQVVAIASNGGGKQALETVQRLLPVLCQ
AHGLTPAQVVAIASNGGGKQALETVQRLLPVLCQAHGLTPAQVVAIASNGG
GKQALETVQRLLPVLCQAHGLTPAQVVAIASNNGGKQALETVQRLLPVLCQ
AHGLTPDQVVAIASNNGGKQALETVQRLLPVLCQAHGLTPDQVVAIASNIGG
KQALETVQRLLPVLCQAHGLTPDQVVAIASNNGGKQALETVQRLLPVLCQA
HGLTPDQVVAIASNGGGKQALETVQRLLPVLCQAHGLTPDQVVAIASNIGGK
QALETVQRLLPVLCQAHGLTPDQVVAIASNNGGKQALETVQRLLPVLCQAH
GLTPDQVVAIASNIGGKQALETVQRLLPVLCQAHGLTPDQVVAIASNIGGRPA
LESIVAQLSRPDPALAALTNDHLVALACLGGRPALDAVKKGLG*GS*AIPVKRG
ATGETKVFTGNSNSPKSPTKGGC*SGGS*TNLSDIIEKETGKQLVIQESILMLPEEV
EEVIGNKPESDILVHTAYDESTDENVMLLTSDAPEYKPWALVIQDSNGENKIK
ML*

ATP8-DdCBE: Left-SOD2 MTS-3×HA-mitoTALE-G1397-DddA$_{tox}$-N-1×UGI
MALSRAVCGTSRQLAPVLGYLGSRQKHSLPDYPYDVPDYAGYPYDVPDYAG
YPYDVPDYAMDIADLRTLGYSQQQQEKIKPKVRSTVAQHHEALVGHGFTHA
HIVALSQHPAALGTVAVKYQDMIAALPEATHEAIVGVGKQWSGARALEALL
TVAGELRGPPLQLDTGQLLKIAKRGGVTAVEAVHAWRNALTGAPLNLTPDQ
VVAIASNIGGKQALETVQRLLPVLCQAHGLTPDQVVAIASNGGGKQALETVQ
RLLPVLCQAHGLTPAQVVAIASNGGGKQALETVQRLLPVLCQAHGLTPAQV

VAIASNIGGKQALETVQRLLPVLCQAHGLTPDQVVAIASNIGGKQALETVQRL
LPVLCQAHGLTPDQVVAIASNIGGKQALETVQRLLPVLCQAHGLTPDQVVAI
ASHDGGKQALETVQRLLPVLCQAHGLTPAQVVAIASNIGGKQALETVQRLLP
VLCQAHGLTPDQVVAIASHDGGKQALETVQRLLPVLCQAHGLTPAQVVAIAS
NIGGKQALETVQRLLPVLCQAHGLTPDQVVAIASNIGGKQALETVQRLLPVL
CQAHGLTPDQVVAIASNIGGKQALETVQRLLPVLCQAHGLTPDQVVAIASH
DGGKQALETVQRLLPVLCQAHGLTPAQVVAIASNGGGKQALETVQRLLPVL
CQAHGLTPDQVVAIASNIGGKQALETVQRLLPVLCQAHGLTPDQVVAIASNG
GGRPALESIVAQLSRPDPALAALTNDHLVALACLGGRPALDAVKKGLG*GS*GS
YALGPYQISAPQLPAYNGQTVGTFYYVNDAGGLESKVFSSGGPTPYPNYANA
GHVEGQSALFMRDNGISEGLVFHNNPEGTCGFCVNMTETLLPENAKMTVVPP
EG*SGGS*TNLSDIIEKETGKQLVIQESILMLPEEVEEVIGNKPESDILVHTAYDES
TDENVMLLTSDAPEYKPWALVIQDSNGENKIKML*

ATP8-DdCBE: Right-COX8A MTS-3×FLAG-mitoTALE-G1397-DddA$_{tox}$-C-1×UGI
MASVLTPLLLRGLTGSARRLPVPRAKIHSLDYKDHDGDYKDHDIDYKDDDD
KMDIADLRTLGYSQQQQEKIKPKVRSTVAQHHEALVGHGFTHAHIVALSQHP
AALGTVAVKYQDMIAALPEATHEAIVGVGKQWSGARALEALLTVAGELRGP
PLQLDTGQLLKIAKRGGVTAVEAVHAWRNALTGAPLNLTPDQVVAIASNIGG
KQALETVQRLLPVLCQAHGLTPDQVVAIASNGGGKQALETVQRLLPVLCQA
HGLTPAQVVAIASNNGGKQALETVQRLLPVLCQAHGLTPDQVVAIASNNGG
KQALETVQRLLPVLCQAHGLTPDQVVAIASNNGGKQALETVQRLLPVLCQA
HGLTPDQVVAIASHDGGKQALETVQRLLPVLCQAHGLTPDQVVAIASNGGG
KQALETVQRLLPVLCQAHGLTPDQVVAIASNGGGKQALETVQRLLPVLCQA
HGLTPDQVVAIASNGGGKQALETVQRLLPVLCQAHGLTPDQVVAIASNNGG
KQALETVQRLLPVLCQAHGLTPDQVVAIASNNGGKQALETVQRLLPVLCQA
HGLTPDQVVAIASNGGGRPALESIVAQLSRPDPALAALTNDHLVALACLGGR
PALDAVKKGLG*GS*AIPVKRGATGETKVFTGNSNSPKSPTKGGC*SGGS*TNLSDII
EKETGKQLVIQESILMLPEEVEEVIGNKPESDILVHTAYDESTDENVMLLTSDA
PEYKPWALVIQDSNGENKIKML*
